# Sensitivity Analysis of Emission Models of Parcel Lockers vs. Home Delivery Based on HBEFA

**DOI:** 10.3390/ijerph18126325

**Published:** 2021-06-11

**Authors:** Maren Schnieder, Chris Hinde, Andrew West

**Affiliations:** The Wolfson School of Mechanical, Electrical and Manufacturing Engineering, Loughborough University, Loughborough LE11 3TU, UK; c.j.hinde@lboro.ac.uk (C.H.); a.a.west@lboro.ac.uk (A.W.)

**Keywords:** last-mile delivery, city logistics, emissions, pollution, collection and delivery point, parcel locker, HBEFA, PM_10_, non-exhaust emissions, cold-start emissions

## Abstract

Global concerns about the environmental effects (e.g., pollution, land use, noise) of last-mile deliveries are increasing. Parcel lockers are seen as an option to reduce these external effects of last-mile deliveries. The contributions of this paper are threefold: firstly, the research studies simulating the emissions caused by parcel delivery to lockers are summarized. Secondly, a demand model for parcel deliveries in New York City (NYC) is created for 365 days and delivery trips to lockers and homes are optimized for 20 “real-world” scenarios. Thirdly, using the emission factors included in the HandBook Emission Factors for Road Transport (HBEFA) database, the maximum percentage of customers who could pick up a parcel by car from parcel lockers that would result in fewer total emissions (driving customers + walking customers) than if home deliveries were adopted is calculated for various pollutants and scenario assumptions (i.e., street types, temperature, parking duration, level of service and vehicle drivetrain). This paper highlights how small changes in the calibration can significantly change the results and therefore using average values for emission factors or only considering one pollutant like most studies may not be appropriate.

## 1. Introduction

### 1.1. The Problem of Last-Mile Delivery

Air pollution is a critical societal concern due to the adverse effects on human health. Particularly in cities, residents are exposed to air pollution levels which are considered dangerous [1]. The European Environment Agency (EEA) names particulate matter (PM), NO_2_ and ground-level O_3_ as the most harmful pollutants to human life [2]. The logistics industry [3], and especially last-mile delivery, is one of the causes of air pollution especially in residential areas [4,5,6] and the city center [7,8], which increases the interest in alternative delivery options.

Parcel lockers are seen as a solution for failed deliveries [9,10,11,12,13], for problems in finding addresses [14] and for return deliveries [14]. Parcel lockers also increase the consolidation of deliveries [7,9,12,15,16,17], decrease the number of drop-off points [16], decrease the vehicle kilometer traveled (VKT) [14,18,19] and decrease delivery cost [8,9,13,20,21]. From an environmental viewpoint, authors have stated that parcel lockers are more sustainable [22], and reduce exhaust emissions [6,8,12,17,23,24], transport-related noise [6] and traffic jams [6]. Only Arnold et al. [25] concluded that, based on a simulation study, parcel lockers reduce the operational costs but increase the external costs.

McLeod et al. [26], Song et al. [21], Hoffer et al. [27], Rai et al. [28] and Liu et al. [4] report that cars are used to pick up parcels from a Collection Delivery Point (CDP) in 43%, 48%, 44.5%, 47%, and 70% of the trips, respectively. Additionally, 50% [4] and 30.6% [28] of the parcel pick-up trips were roundtrips to the CDP (i.e., not integrated into a trip chain). Of those who chained their pick-up trips with other trips, 52.8% made a detour [28]. So, these figures indicate that the environmental effect of these pick-up trips can be substantial [4].

However, according to Liu et al. [4], most studies ignore the external effects of picking up a parcel from a CDP. Some authors state that customers are inclined to use non-motorized modes of transport to pick up parcels or the pick-up trip would not increase the distance travelled (e.g., [6,8,19]). As shown in Table 1, almost no published study considers pick-up trips by customers and assumes that first delivery attempts are made to lockers. Only two studies consider emissions, customer trips and first-attempt deliveries to lockers. However, one of these includes the customer trips only in the sensitivity analysis and not as part of the main simulation [22]. Most of the studies which consider the trips by customers compare the effect of returning a failed delivery to a depot with delivering these to a nearby CDP. Table 1 is limited to studies where parcels are delivered by a company and not by crowd shipping given that the delivery methods are dissimilar. Studies that evaluated the emissions of public transport based crowd shipping are reported in [29,30].

Jiang et al. [8], Carotenuto et al. [19], Saad et al. [31] and a study by the Department of Robotics and Mechatronics at the AGH University of Science and Technology in Krakow (Poland) (reported in [6,7,17,32]) compared the emissions produced by home delivery with delivery to parcel lockers as a first attempt.

Jiang et al. [8] proposed a traveling salesman algorithm to minimize carbon emissions for delivery to customer homes and parcel lockers. Depending on the willingness of customers to travel to the locker, they concluded that the carbon emissions can be reduced by 18.7–51.2%. However, they ignore the potential emissions generated by the customer stating that “In reality, customers prefer to walk to PLs within 500 m to pick up their parcels” [8], (p. 61625). Furthermore, only straight-line distances are used. They limited the study to only 81 customers and simulated a relatively small area of just 1.3 km^2^ with 10 parcel lockers.

Carotenuto et al. [19] focused on solving a Multi-Depot Capacitated Vehicle Routing Problem (MDCVRP) and therefore ignored the trips by customers. The simulated CO_2_ emissions of delivery to lockers was 21% lower than those resulting from delivery to homes. The study included comparably large stem miles. Based on the map, the stem miles are larger than double the diagonal of the delivery area.

Saad et al. [31] assumed that the delivery vehicles would only pollute 0.00028324 kg of CO_2_ per km and considered an average velocity of 30 m/h. However, the estimated average speed of a delivery van in London including the time spent driving and walking is 3 km/h (i.e., 100-fold larger) (own calculation based on the results reported in [36]) and typical emissions of vehicles usually range from 0.1 to 0.3 kg/km (i.e. 300 to 1000-fold larger). In contrast with Jiang et al. [8] and Carotenuto et al. [19], Saad et al. [31] considered that a delivery vehicle can transport more parcels when delivering to parcel lockers than home delivery due to the reduced delivery time.

Iwan et al. [7], Lemke et al. [17], Moroz et al. [6], and Kiousis [32] reported the results of a study conducted by the Department of Robotics and Mechatronics at the AGH University of Science and Technology in Krakow (Poland), which compares real delivery trips to customer homes with real delivery trips to parcel lockers. All of the four papers concluded that delivery to parcel lockers produces only 5% of the emission produced by home deliveries. They state that, on average, 60 parcels were delivered in a home delivery shift, while 600 parcels were delivered per shift to parcel lockers. However, it is unknown whether the deliveries serve the same area. If the home delivery trips cover the entire city area, while trips to lockers only cover the dense central area, it would be an unfair comparison. Additionally, 60 parcels per shift appears low for a dense area as it is only one-third of the average number of parcels delivered per shift in London [36].

Giuffrida et al. [22] assumed that parcels are either delivered to a parcel locker and stored for three days or three home delivery attempts are made. If the parcel has not been picked up from the locker or all three of the home delivery attempts fail, the parcel will be returned to the depot. Only the customer trips to the depot are considered but not to the locker in the main simulation. Thus, they conclude that the emissions caused by the customer are significantly lower than those of the courier as customer trips only occur when a regular delivery fails. Home delivery produced 0.299 kg CO_2_e per parcel and delivery to lockers produced 0.102 kg CO_2_e per parcel. In the sensitivity analysis, they determined that the customer can only deviate by less than 0.94 km from their usual route in urban areas and 6 km in non-urban areas before home delivery becomes a better option.

Kiousis et al. [33] concluded, based on a simulation in PTV VISSIM, that parcel lockers offer great benefits for the delivery company and the municipality. However, the table in their results section shows, for example, that 1,118,742 customer vehicles were required to pick up the parcels while the baseline scenario only required 5 vans. This seems to be a printing error. They present minimal reductions in the emissions (i.e., −0.40% to −0.30%), while the VKT increases significantly (i.e., home delivery: 58,097 m; parcel locker: 5265 m (van) + 118,742 m (customer)). The reduction in the emissions can only be explained by their assumption that 49% of customers are not driving and it may be assumed that customer vehicles produce less emissions per km.

While the previously mentioned studies are focused on delivering to parcel lockers and homes as a first attempt, the following three studies are focused on delivering failed deliveries to either the depot or a CDP. Generally, the studies assume that a courier will at first attempt to deliver the parcel to the customer’s home and if this fails, will either deliver the parcel to a CDP or return it to the depot. Thus, these studies are essentially comparing: (i) multiple home delivery attempts and a trip by the customer to the depot, with (ii) one home delivery attempt, a drive to a CDP by the delivery vehicle and a customer picking up the parcel from this CDP. Thus, the potential advantages of the reduced VKT, which can be seen when the delivery to lockers has been made as a first attempt, is not considered in this research.

Song et al. [21] assumed that the courier will at first attempt to deliver a parcel to the customer’s home and if that is unsuccessful will either deliver it to a CDP or a depot. They estimated the delivery cost for various combinations of failed deliveries, CDP locations and percentages of customers traveling to the depot ranging from 10% to 50%. They calculated the CO_2_ emissions only for one of these scenarios and depending on the density of CDPs, the CO_2_ emissions can be reduced by up to 40%. Song et al. [21] acknowledged that a large percentage of customers need to walk to a CDP in order for the reduction in emissions to be achieved.

The study by Song et al. [34] reported the CO_2_ emissions for percentages of failed deliveries (varying from 0% to 50%) and number of CDPs (varying from 4 to 139) in two different cities. If the percentage of failed deliveries is either 10% or 20%, redelivery to the customers was better than delivery to CDPs as redeliveries can be optimized into the order of deliveries whereas delivery to CDPs happens only after all parcels in the area have been delivered to customers.

Edwards et al. [35] calculated the CO_2_ emissions for three options to handle failed deliveries: (i) redelivery, (ii) delivery to depot, and (iii) delivery to a CDP. They did not simulate individual parcel delivery trips. Instead, they only use average values (e.g., one delivery trip length (i.e., 50 miles), one number of parcels per trip (i.e., 120), and four distances between customers and depots). However, in contrast with other studies, they consider that the customer might choose public transport to pick up parcels. They concluded that CDPs are always the better option given that customers travel a shorter distance.

### 1.2. Description of the Study and Contribution

In contrast with the published studies, the research outlined in this paper considers the emissions of the customers as well as the delivery vehicles and assumes that first delivery attempts are either made to a parcel locker or the customer’s home. While all of the studies mentioned in the literature review only consider CO_2_ emissions or CO_2_ equivalents, this study considers five key emission parameters and their variations (i.e., PM_10_ (exhaust), PM_10_ (non-exhaust), CO_2_, and CO_2_e (WTW) [37]). The value of considering only CO_2_ is limited given that PM_10_, NO_2_ and ground-level O_3_ but not CO_2_ are named as the most harmful pollutants to human life by the European Environment Agency (EEA) [2]. Furthermore, Well-to-Wheel (WTW) and non-exhaust emissions are important to account for drivetrain types where most of the emissions are not produced by the engine but rather by the tires or by the production of the energy such as electric cars. The increased non-exhaust emissions of electric vehicles caused by their heavier weight can result in higher total (i.e., exhaust + non-exhaust) PM_2.5_ and PM_10_ emissions [38]. All papers listed in Table 1 fail to acknowledge that the emissions vary depending on the gradients, level of service (LOS, i.e., traffic flow), engine types, vehicle ages, cold start/warm start (i.e., customer trip length, prior parking duration), street types and temperatures.

The research contribution of this study is threefold: firstly, this study reviews published literature comparing home delivery and delivery to lockers focusing on emissions. Secondly, this study shows that the sustainability of delivery to lockers depends on the mode the customers choose to pick up the parcels and the operating area. Thirdly, it is shown how variations in the parameters in the emission model (e.g., temperature, street types, drivetrain type) and the selection of pollutants (e.g., PM_10_, NO_x_) affect the results. To achieve this, this study compares 20 scenarios of last-mile deliveries in New York City (NYC) each simulated 365 times.

## 2. Methods

### 2.1. Parcel Delivery Simulation

The dataset of potential parcel recipients has been estimated based on a dataset of all addresses [39], and the population density in NYC [40]. A binomial random number generator in the Python programming language (n = number of residents at an address, p = probability that a resident receives a parcel) [41] has been used to create a list of customers receiving a parcel for each day of the simulation. The probability that a resident receives a parcel has been estimated based on a survey [42] of the number of parcels received by residents in NYC based on their neighborhood. Only non-food-based deliveries have been included in this study. Each address has its deliveries assigned to the closest parcel locker. It is assumed that customers choose to travel to the closest CDP. The bike-sharing stations of Citi Bike NYC [43] have been used as representative locations of parcel locker stations due to their continent location and easy access for pedestrians and cyclists.

In total 20 scenarios have been simulated (Table 2).

Thus, each simulation run covers an area approximately 0.55 km further to both the east and west and approximately 0.7 km further to both the north and south than the next smaller one (1). The center point is at latitude = 40.764940 degrees, longitude = −73.977080 degrees near the amazon fulfilment center in Manhattan.

(1) $$40.764940-\left(0.00457*\left(s+3\right)\right)<longitude_{s}<40.764940+\left(0.00457*\left(s+3\right)\right)$$

$$-73.977080-\left(0.00455*\left(s+3\right)\right)>latitude_{s}>-73.977080+\left(0.00455*\left(s+3\right)\right)$$

0≤s<20

Each of the 20 operating areas is simulated for 365 days. For each day, 175 customers have been randomly selected to receive a parcel. For home delivery, an additional 25 randomly selected parcels are added to the delivery trip to account for failed deliveries. Hence, 25 of the 200 home delivery attempts are assumed to fail, while all 175 deliveries to lockers are successful. Other authors suggested that failed deliveries account for approximately 10% [44], 18.8% [11], 15% [45], 12% [26] or 10–50% [21,35] of all deliveries. The failed delivery rates vary due to the variation in policies for handling failed deliveries by couriers [35]. Note: 200 deliveries represent the 0.77 percentile of the number of parcels handled (i.e., failed and successful deliveries and pick-up trips of parcels) per tour for a delivery company in London (own calculation based on [36]).

### 2.2. Delivery Tour Distance and Customer Travel

A locally hosted Open-Source Routing Machine (OSRM) [46] using Open Street Map (OSM) street network data [47] has been used to optimize the order of the parcel drop-off points and to calculate the travel distance. Even though 200 delivery attempts are made for home delivery, it is assumed that only 175 parcels are delivered successfully to account for failed deliveries. Thus, the distance per parcel is calculated by dividing the total distance by the number of successful deliveries. The travel distance only includes the round-trip distance between all delivery points and no stem miles are included as it is assumed that the parcels will be delivered by van from a centrally located warehouse. For example, the Amazon Fulfillment Center, 6 W 35th St, New York, NY 10001, United States, is close to the center of the operating areas. The distance customers travel is calculated based on the roundtrip distance by car between the customers’ homes and locker for every delivered parcel.

### 2.3. Emission Modeling—HBEFA

Handbook Emission Factors for Road Transport (HBEFA 4.1) is an emission factor database, which can be used to estimate emissions. It provides emission factors in g veh km^−1^ for a variety of vehicle categories, drivetrains, traffic situations, cold and warm start, and climatic conditions [37]. The emissions factors considered in this study are total hydrocarbons (HC), carbon monoxide (CO), nitrogen oxides in NO_x_ equivalents (NO_x_), particulate matter (PM_10_), PM_10_ caused by non-exhaust emissions such as tire wear (PM_10_ (non-exhaust)), reported carbon dioxide excluding biofuel share (CO_2_ (rep)), total carbon dioxide including biofuel share CO_2_ (total), CO_2_ equivalents containing CO_2_, CH_4_ and N_2_ (CO_2_e), CO_2_ equivalents of Well-to-Wheel emissions (CO_2_ e WTW) [37]. HBEFA has been chosen given that it is one of the most frequently used models and includes various traffic situations [48]. Models such as COPERT, which define emission factors as a function of average speed, are not appropriate for this study given their limited calibration options. Models which consider variable traffic situations such as TEE could not be used in this study given that most of the required calibration options (e.g., signal settings, queue length) are unknown in this simulation.

HBEFA is not calibrated for the use in American cities. Hence, an emission model specific to Germany has been adopted to ensure that the changes in the model are calibrated correctly. It was not the goal of this research to exactly quantify the amount of emissions but rather to conduct a sensitivity analysis of the emission model to emphasize that even small changes in the calibration of the model can significantly change the results. Thus, it is not important whether the emission model is calibrated specifically for NYC. It is more important that the emission model itself has been tested and validated. The default fleet composition is representative of the vehicles registered in Germany in 2020. The default traffic situation parameters in this study are urban, primary-city non-motorway, maximum speed of 50 km/h, free flow and 0% gradient. The temperature, trip duration, Well-to-Tank (WTT) emissions and parking duration is representative of the German average for light commercial vehicles (LCV) and private motor vehicles (PMV).

Instead of calculating the emissions for locker and home delivery, this study reports the maximum mode share, which is defined as the maximum share of customers who can pick up a parcel by car before home delivery becomes the better option in terms of emission generation. The maximum mode share is calculated as follows:

(2) $$m_{c}=min\left\{\frac{e_{v}\left(d_{H}-d_{L}\right)}{e_{cw}*d_{c}+e_{cc}*f_{c}},1\right\}$$

where

$m_{c}$ Maximum mode share for private motor vehicles,
$e_{v}$ Emission factors of light commercial vehicles (warm),
$e_{cw}$ Emission factors of passenger vehicles (warm),
$e_{cc}$ Cold-start emission factors of passenger vehicles,
$f_{c}$ Share of the parcel pick-up trip of the total trip length (customer),
$d_{H}$ Distance per parcel by delivery van (home delivery),
$d_{L}$ Distance per parcel by delivery van (locker delivery), and
$d_{c}$ Roundtrip distance between customer and locker.

For some of the simulations in this paper, HBEFA is not differentiating between hot- and cold-start emissions and therefore $e_{cc}$ is set to 0 in these cases. For changes in the temperature, parking and trip duration, HBEFA supplies the hot- and cold-start emissions separately. The factor $f_{c}$ is 1 in all cases except in the variation of the total trip length. For the variation of the total trip length only the corresponding share of the cold-start emissions has been considered for the parcel pick-up trip. In other words, if the total trip is twice as long as the parcel pick-up trip, only half of the cold-start emissions are attributed to the parcel pick-up trip.

The figures in this paper allow for a mode share for cars to be larger than 100% to emphasize the sensitivity of the results. Hence, the following equation has been used:

(3) $$m_{c}=\frac{e_{v}\left(d_{H}-d_{L}\right)}{e_{cw}*d_{c}+e_{cc}}$$

### 2.4. Limitations

This simulation assumes that the driver stops right in front of the coordinates of each address and not in a nearby parking spot. The walking distance between the parking spot and customer homes accounts for 40% of the roundtrip distance and 62% of the total round-trip time in central London [49]. This has been ignored as no data on parking locations/restrictions were available for NYC. While the walking distance from the parking spot to the customer in central London and Manhattan might be similar, the outer areas in the larger operating areas are mainly family homes, which should allow drivers to park in front of the address. Given that delivery companies in NYC pay, on average, $750 per truck [50] or $1394 per driver [51] per month in parking fines, it can be assumed that drivers in NYC prefer to park close to the customer’s address instead of driving to a close-by parking spot.

The simulation only considers the delivery of parcels but not the pick-up of returned parcels. The failure rate of parcels picked up from the customer’s home might be similar to the percentage of failed deliveries. However, it is possible that lockers might be full or defect when customers want to use a locker to return their parcel. Hence, the failure rate of 0% for deliveries to parcel lockers cannot be applied to customers dropping of parcels.

## 3. Results

### 3.1. Distance

**Figure 1:**
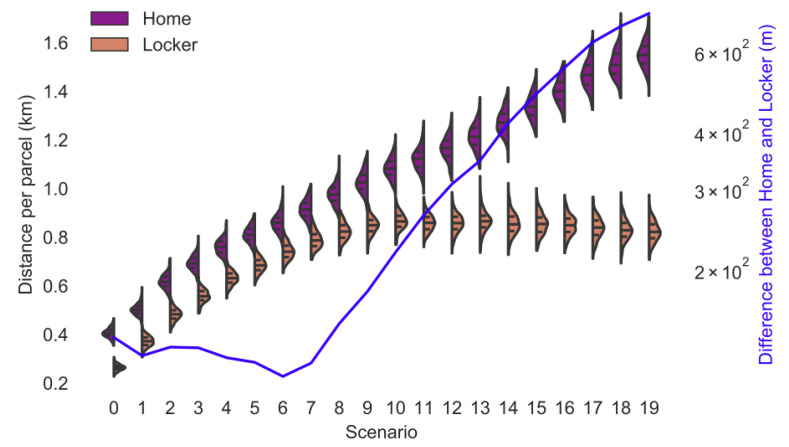
Distance per parcel for delivery to lockers (delivery van) and customer’s homes as well as the difference between both delivery types.

Figure 1 shows the distance per parcel for home delivery and delivery to lockers for each operating area. The distance for delivery to lockers levels out given that lockers are assumed to be only positioned in central areas of NYC to account for the fact that there might be limited numbers of lockers located in rural areas. Hence, the customers living outside the parcel locker network pick up the parcel from a parcel locker at the edge of the parcel locker network. The more customers live outside the parcel locker network, the more parcels are delivered to the lockers at the edge of the parcel locker network, which reduces the number of lockers the driver has to drive to. Hence, the distance per parcel for delivery to parcel lockers has a peak at operating area 10 and reduces slightly in larger operating areas.

The difference between the distance travelled per parcel for home delivery and delivery to parcel locker has a slight decreasing trend until operating area 7 due to the reduction in the number of parcels delivered to the same locker as explained before. Therefore, the advantage of reducing the number of drop off points by using parcel lockers is decreasing. The difference is increasing in operating areas larger than 7 as explained before.

**Figure 2:**
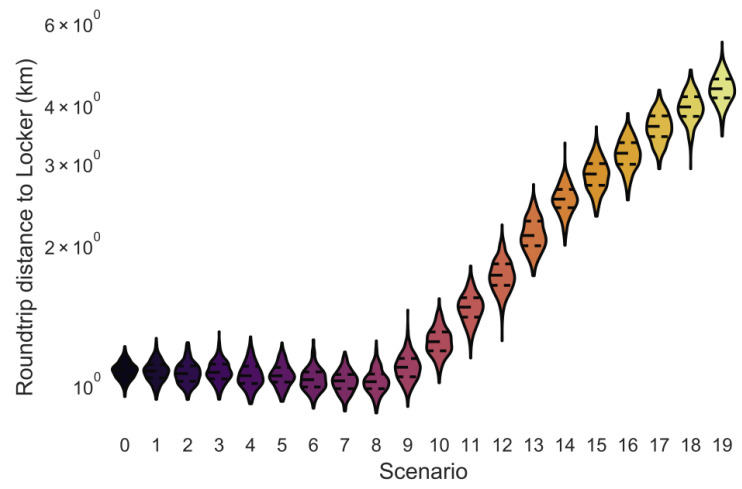
Average roundtrip distance to between lockers and customer’s homes.

The round-trip distance customers travel to parcel lockers is low until operating area 8 given that the number of customers living outside the parcel locker network area is insignificantly small (Figure 2). The roundtrip distance in operating area 0 is larger than in operating area 8 due to one-way streets and turn restrictions in central Manhattan. The share of customers living outside the parcel locker network area is constantly increasing for larger operating areas.

The chosen distribution of parcel lockers in this research is similar to the spatial distribution of parcel lockers in Germany. In this simulation, the round-trip distance in operating area 0 to operating area 9 is approximately 1 km (Figure 2), which is similar to the one-way distance of 600 m for residents in German cities [9]. In larger operating area, the percentage of customers living outside the area with parcel lockers is increasing and the average roundtrip distance is increasing to 4.3 km in operating area 19, which is similar to rural parts of Germany, where residents live, on average, 3 km away (one-way) from parcel lockers [9].

### 3.2. Average Case for All Emissions

**Figure 3:**
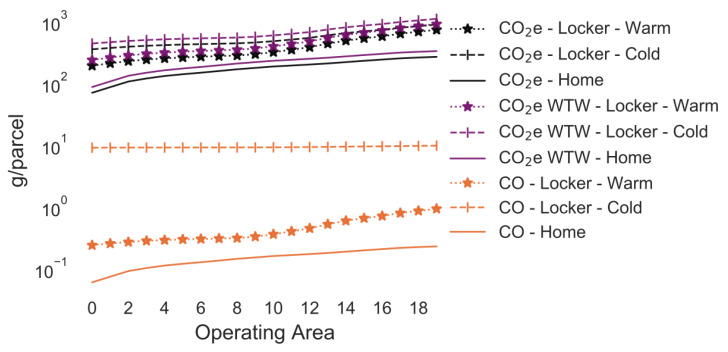
Selected pollutants for home delivery and delivery to lockers.

**Figure 4:**
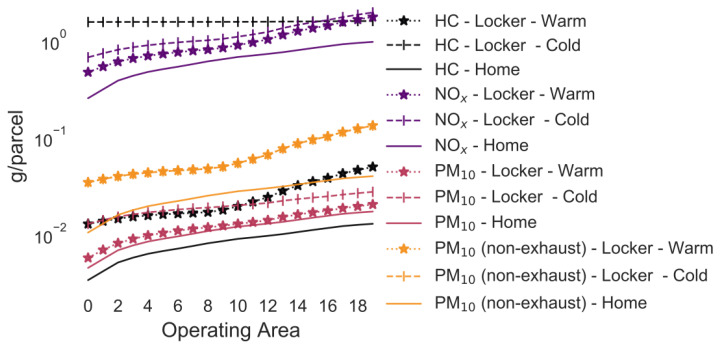
Selected pollutants for home delivery and delivery to lockers (logarithmic scale).

In the following example, every customer drives a car to pick up a parcel. The traffic situation is “URB/Trunk-City/50/Freeflow”, with 0% gradient and a 2020 vehicle fleet. The average temperatures and parking duration in between trips representative of Germany have been used. The customer’s trip to the parcel locker is either assumed to be part of a trip of average length in Germany (i.e., proportionate consideration of cold-start emissions) or is a separate round trip (i.e., full consideration of cold-start emissions). Figure 3 and Figure 4 shows the total emissions of customers and delivery van combined. The figures compare the effect of cold start (i.e., the parcel pick-up trip is the only trip taken by the customer) and warm start (i.e., the customer integrated the pick-up trip into a larger trip) as well as home delivery vs. delivery to parcel lockers. Delivery to parcel lockers causes CO_2_ emissions per parcel that are approximately twice as high than those for home delivery for every operating area when the customer picks up the parcel with a car that has a warm engine (Figure 3). For the cold start, it is assumed that the vehicle has been parked for more than 12 h before the trip to the locker and the CO_2_ emissions per parcel are almost 3-fold as high for lockers than for home delivery.

The HC and CO emissions are 3-fold higher for deliveries to lockers compared with home deliveries under warm start conditions, but more than 208-fold and 68-fold, respectively, larger for lockers during cold start (Figure 3 and Figure 4). The effect of cold start is not as extreme for NO_x_, PM_10_. PM_10_ (non-exhaust) emissions are not affected by the cold-start emissions. The emissions of these pollutants are approximately 1–3-fold (warm start) and 2–5-fold (cold start) larger for delivery to lockers than home delivery.

In the following scenarios, one of seven parameters (e.g., gradient, temperature, trip length, street type) of the emission model is varied, while all others are assumed to be as described in 3.2.

To compare the emissions produced by both delivery options, the maximum fraction of customers, who can pick up a parcel by car from a locker and would still produce fewer emissions than home delivery, is calculated. In other words, if more customers would drive to pick up a parcel, home delivery would be better. If fewer customers drive, delivery to parcel lockers is better. This fraction is referred to as maximum mode share hereafter.

### 3.3. Overview: Variation in the Emission Model

**Figure 5:**
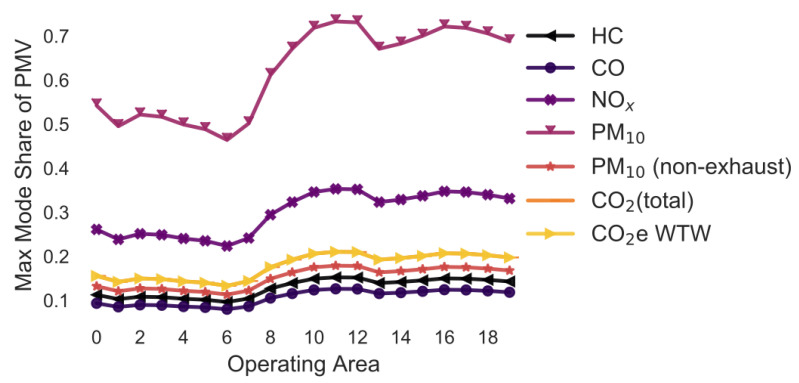
Maximum mode share of customers who can pick up a parcel by car (varying street type) (note: CO_2_ (total) and CO_2_e WTW are the same).

**Figure 6:**
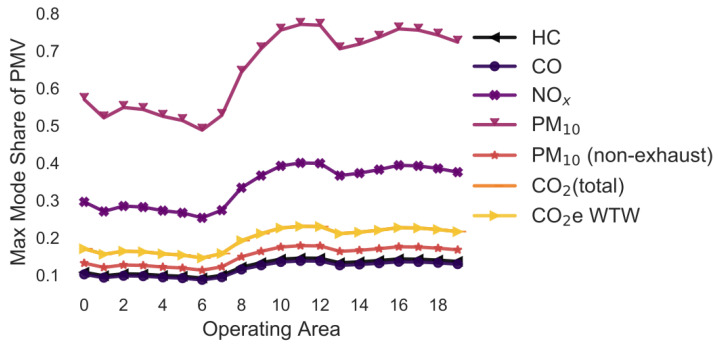
Maximum mode share of customers who can pick up a parcel by car (varying gradient) (note: CO_2_ (total) and CO_2_e WTW are the same; HC and CO are the same).

**Figure 7:**
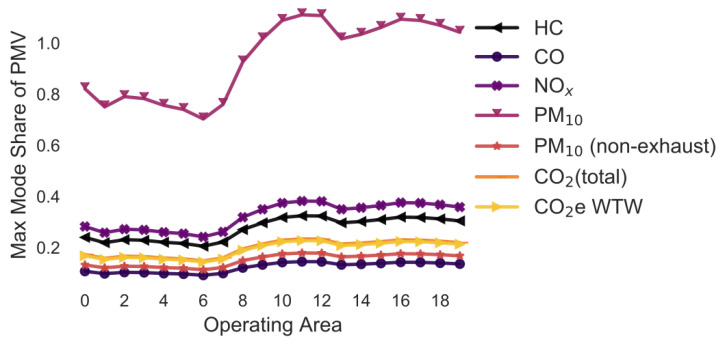
Maximum share of customers who can pick up a parcel by car (varying vehicle fleet age) (note: CO_2_ (total) and CO_2_e WTW are the same).

**Figure 8:**
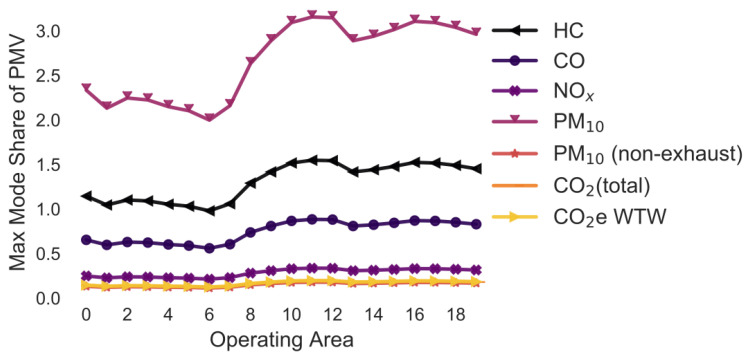
Maximum share of customers who can pick up a parcel by car (varying delivery vehicle stock age) (note: CO_2_ (total), CO_2_e WTW and PM_10_ (non-exhaust) are the same).

**Figure 9:**
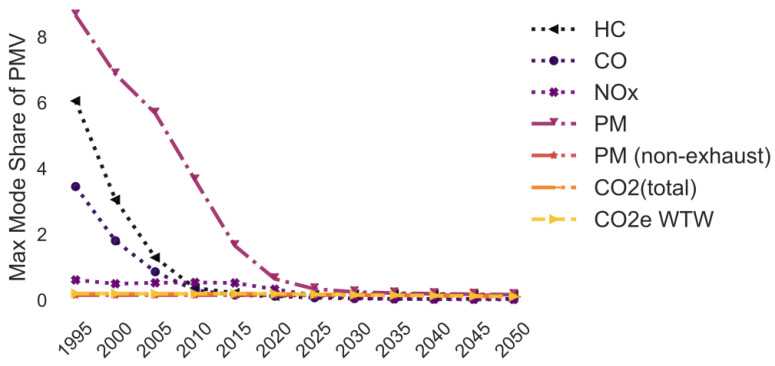
Maximum share of customers who can pick up a parcel by car (average over operating areas, varying delivery vehicle age) (note: PM_10_ (non-exhaust), CO_2_ (total) and CO_2_e WTW are the same).

**Figure 10:**
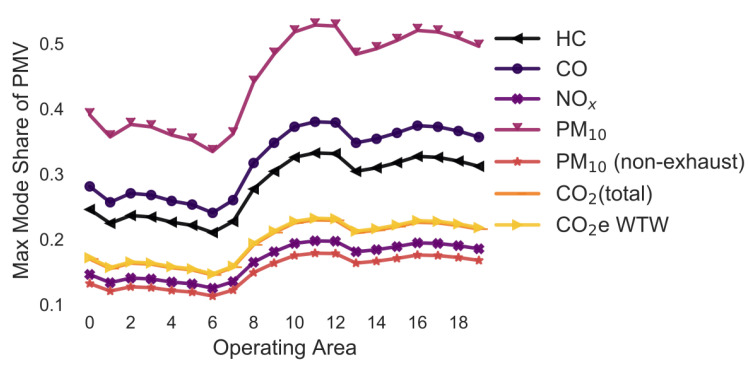
Maximum share of customers who can pick up a parcel by car (varying delivery vehicle drivetrain) (note: CO_2_ (total) and CO_2_e WTW are the same).

**Figure 11:**
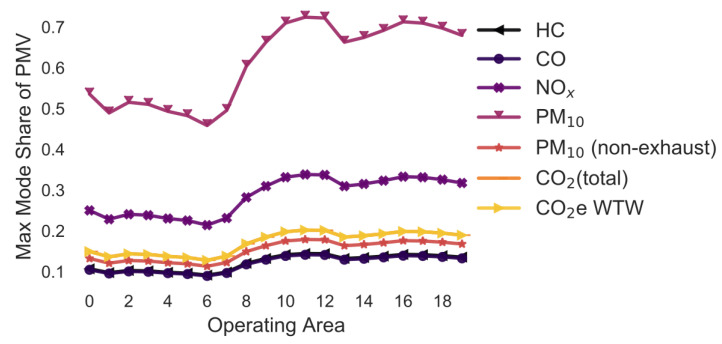
Maximum share of customers who can pick up a parcel by car (varying level of service) (note: CO_2_ (total) and CO_2_e WTW are the same).

**Figure 12:**
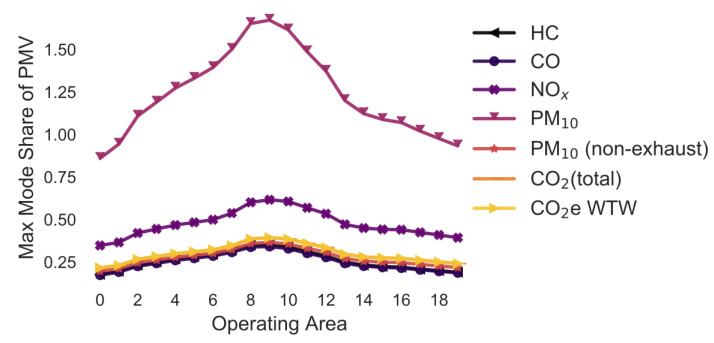
Maximum share of customers who can pick up a parcel by car (varying level of service for home delivery and customer trips).

**Figure 13:**
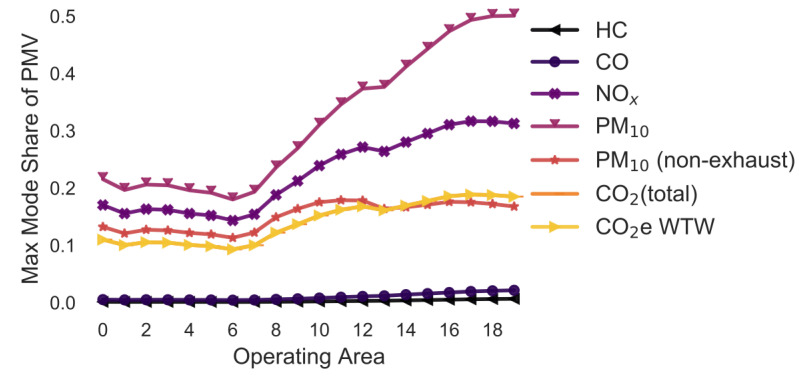
Maximum share of customers who can pick up a parcel by car (varying parking duration before customer trip) (note: CO_2_ (total) and CO_2_e WTW are the same).

**Figure 14:**
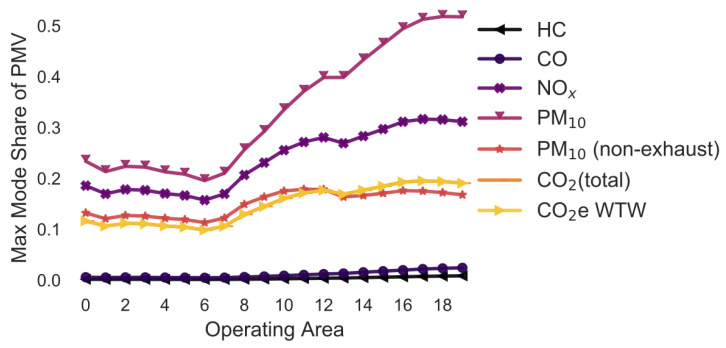
Maximum share of customers who can pick up a parcel by car (varying temperature) (note: CO_2_ (total) and CO_2_e WTW are the same).

**Figure 15:**
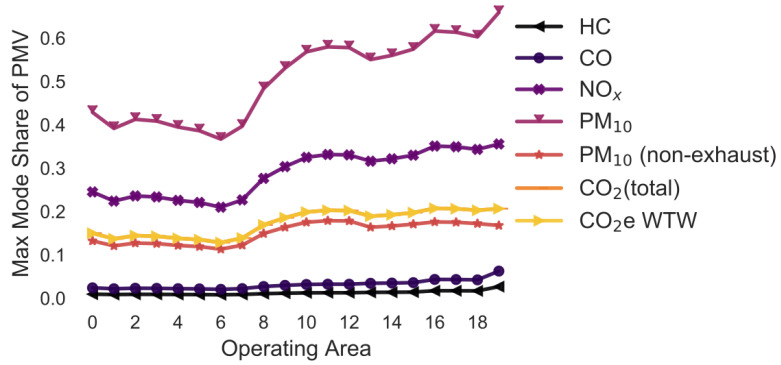
Maximum share of customers who can pick up a parcel by car (varying customer trip length) (note: CO_2_ (total) and CO_2_e WTW are the same).

In the following simulations, the maximum mode share has been calculated for every combination of pollutant, operating area and variation of a selected parameter. For example, in Section 3.2, the maximum mode share is calculated for every combination of pollutant, operating area and gradient (i.e., 0%, +/−2%, +/−4%, +/−6%). To ease the interpretability of the figures, only the means of the maximum mode share for each combination of pollutant and operating area are shown in Figure 5, Figure 6, Figure 7, Figure 8, Figure 9, Figure 10, Figure 11, Figure 12, Figure 13, Figure 14 and Figure 15. By how much the maximum mode share changes due to the variation in the simulation parameter is noted in the text.

The lines in Figure 5, Figure 6, Figure 7, Figure 8, Figure 9, Figure 10 and Figure 11 have a similar trend. While the maximum mode share is overall increasing with the operating area size, it follows a distinct fluctuation: a local minimum for operating area 1, 6, and 13 as well as local maxima around operating area 9 to 12. The first two local minima can be explained by the local minima of the difference between the distance per parcel for delivery to parcel lockers and homes (Figure 1). The distance the customer travels to a parcel locker (i.e., part of the denominator in (2)) stays relatively constant for operating area 1 to 8, while the difference between the distance per parcel for delivery to parcel lockers and homes (i.e., part of the numerator in (2)) have local minimum at operating areas 1 and 6. While both the numerator and denominator are increasing from operating area 12 to 13, the numerator (i.e., difference between the distance per parcel for delivery to parcel lockers and homes) is increasing much less than the denominator (i.e., 1.13 vs. 1.23). Hence, the local minimum at operating area 13. The steep increase in between operating area 7 to 11 is caused by the steep increase in the difference between the distance per parcel for delivery to parcel lockers and homes (approximately 1.2 per operating area), while the distance to parcel lockers starts increasing only from operating area 8 onwards with a lower rate (approximately 1.1 per operating area). In short, the shape of the curves is mainly caused by the relationship and increase in distance the customer travels to parcel lockers (i.e., denominator in (2)) and the difference between the distance per parcel for delivery to parcel lockers and homes (i.e., numerator in (2)).

It should be noted that the mode shares for CO_2_ (total) and CO_2_e WTW are usually the same and hence only one line is visible in the graphs. All the graphs have been produced using the python libraries Matplotlib [52] and Seaborn [53].

### 3.4. Variation in the Street Types and Max Speed (Figure 5)

The following street types and maximum speeds have been considered: Trunk-City/50, Trunk-City/60, Distr/30, Distr/40, Distr/50, Distr/60, Local/30, Local/40, Local/50, Local/60, Access/30, Access/40 and Access/50. All are assumed to be under free-flow conditions in an urban setting.

The lines have a similar shape as described before. If the two delivery types would only be compared based on their PM_10_ emissions, approximately 50–70% of all customers could pick up a parcel from a parcel locker and delivery to lockers would still be better. This is due to the higher emission factor for LCV than PMV. If the comparison would be based on HC, CO, CO_2_, and PM (non-exhaust) emission, less that 20% of customers could drive to pick up a parcel. Changing the street type results into a +/−1.4 pp change in the maximum mode share for the pollutant HC. For CO, this is +/−3.9 pp, NOx +/−5.3, PM_10_ +/−4.2 pp, PM_10_ (non-exhaust) +/−0.0 pp, CO_2_ (total) +/−2.2 pp, and CO_2_e WTW +/−2.2 pp.

### 3.5. Variation in the Gradients (Figure 6)

The simulation is run under the assumption that the maximum gradient is either 0%, +/−2%, +/−4%, or +/−6% (Figure 6). The average maximum mode share in Figure 5 and Figure 6 is similar. The variation in the maximum mode share due to changes in the gradient is much lower with +/−0.4 pp to +/−0.7 pp for all pollutants except for PM_10_ (non-exhaust).

### 3.6. Variation in the Vehicle Age (Figure 7)

In this simulation, the maximum mode share has been calculated for historic and projected fleet compositions in Germany for the years from 1995 to 2050 in 5 years steps (Figure 7). The aim of the simulation is to predict how the maximum mode share will change in the future and has changed in the past.

While the trend of the lines is similar to Figure 5 and Figure 6, the average maximum mode share is approximately 30 pp higher for PM_10_ and 20 pp for HC. The variation in the maximum mode share due to changes in the annual traffic composition is in most cases much larger compared to the previous examples: +/−19.7 pp for HC, +/−11.2 pp for CO, +/−11.1 pp for NO_x_, +/−32.6 pp for PM_10_, +/−0.0 pp for PM_10_ (non-exhaust), +/−2.4 pp for CO_2_ (total), and +/−1.5 pp for CO_2_e WTW.

### 3.7. Variation in the Delivery Vehicle Fleet Age (Figure 8 and Figure 9)

The following simulation is the same as before apart from that the customer always uses a vehicle representative of the fleet composition in Germany in 2020. The aim of the simulation is to estimate how the emissions change if delivery companies update their fleet more or less proactively. The average maximum mode share has the same trend but is higher than the previous scenarios. The average maximum mode share is larger than 1, meaning that even if everyone drives to pick up a parcel, delivery to parcel locker would always be better (Figure 8). Additionally, the variation caused by changes in the delivery vehicle stock is larger than in the previous examples: +/−296.4 pp for HC, +/−170.8 pp for CO, +/−29.7 pp for NO_x_, +/−424.8 pp for PM_10_, +/−0.0 pp for PM_10_ (non-exhaust), +/−4.8 pp for CO_2_ (total), and +/−4.5 pp for CO_2_e WTW.

Figure 9 highlights the large change in emissions of the delivery vehicle fleets over the years. Particularly the PM_10_, HC and CO emissions of delivery vehicles have improved massively over the last 25 years. The maximum mode share of 2020 is only 2–7% of the maximum mode share of 1995 for these pollutants. However, the maximum mode share is predicted to stay rather constant in the following years. Hence, it is not predicted that the emissions of LCVs will reduce as much over the next years compared to before.

### 3.8. Variation in the Delivery Vehicle Drivetrain (Figure 10)

In this simulation, the delivery vehicle drivetrain varies (i.e., petrol, diesel, electricity, biofuel CNG/petrol, plug-in hybrid petrol/electric, and plug-in hybrid diesel/electric) while the customer uses a vehicle representative of the annual traffic composition in Germany in 2020. The results indicate that even if the delivery vehicle is electric, parcel lockers are still a better option based on PM_10_ (non-exhaust) and CO_2_ WTW if less than 15% or 11% of customers pick up a parcel by car, respectively. If all delivery vehicles are diesel vans, NO_x_ and PM_10_ allow for the highest maximum mode share (i.e., 34%, 67%), while for petrol vans HC and CO petrol vehicles allow for the highest maximum mode share (i.e., 50%, 79%). The maximum mode share is less than 20% for all other drive trains and pollutant combinations (Table 3).

The trend of the average of the maximum mode share (Figure 10) is similar to the previous examples.

### 3.9. Variation in the Traffic Flow/Level of Service (LOS) (Figure 11)

Figure 11 shows how variations in the LOS affect the maximum mode share. It is assumed that the same LOS applies to all trips. The street type is an urban road (trunk-city) with a maximum speed of 50 km/h. The LOS ranges from free flow (~49 km/h), heavy (~38 km/h), saturated (~24 km/h), stop and go (~12 km/h), to stop and go2 (~6 km/h). Like in the previous examples, PM_10_ allows for the largest maximum mode share of approximately 45–75%. NO_x_ allows for a mode share of 20–35%. The maximum mode share is less than 20% for all other pollutants.

The change in the maximum mode share due to variation in the LOS is +/−0.5 pp for HC, +/−1.5 pp for CO, +/−4.5 pp for NO_x_, +/−4.0 pp for PM_10_, +/−0.0 pp for PM_10_ (non-exhaust), +/−2.0 pp for CO_2_ (total), and +/−2.0 pp for CO_2_e WTW.

### 3.10. Variation in the LOS Assuming That Lockers Are Always Delivered under Free-Flow Traffic Conditions (Figure 12)

The delivery to locker happens under free-flow conditions (i.e., night time) and the LOS for delivery to homes and customer travel varies. While delivery to lockers at night might offer advantages by potentially reducing the emissions, night time delivery causes other environmental problems such as noise [54].

The variation in the maximum mode share due to changing the traffic condition for home delivery and the customer pick-up trips is larger compared to most of the previous examples: +/−13.8 pp for HC, +/−14.7 pp for CO, +/−14.8 pp for NO_x_, +/−64.9 pp for PM_10_, +/−10.2 pp for PM_10_ (non-exhaust), +/−11.8 pp for CO_2_ (total), and +/−11.8 pp for CO_2_e WTW. The results indicate that the maximum mode share is in some cases (e.g., PM_10_) larger than 1, which means that delivery to lockers is always better than home delivery even if every customer uses a car to pick up their parcel (Figure 12). The overall trend also differs from the previous examples, given that maximum mode share has a peak at operating area 9.

### 3.11. Variation in the Parking Duration between Customer Trips (Figure 13)

The time a customer’s vehicle is parked before a trip ranges from 0 to 1 h to more than 12 h in 1 h steps in this simulation. The trend of the average maximum mode share is different due to the effect of cold-start emissions. The cold-start emissions increase the emissions of shorter customer trips more than for larger trips. The maximum mode share for all pollutants is less then 25% for operating areas 0 to 7 given that the customer travel distance is small and relatively constant for operating areas 0 to 7 (Figure 2). The overall increase in the maximum mode share from operating areas 7 onwards is stronger given that the effect of the cold-start emissions per km is reduced due to the increase in the customer trip length. The variation in the maximum mode share due to changes in the parking duration before customer trips is rather small: +/−0.3 pp for HC, +/−1.3 pp for CO, +/−2.4 pp for NO_x_, +/−11.6 pp for PM_10_, +/−0.0 pp for PM_10_ (non-exhaust), +/−3.7 pp for CO_2_ (total), and +/−3.7 pp for CO_2_e WTW.

### 3.12. Variation in the Temperature (Figure 14)

The temperature of the simulations has been varied from −10 to +25 °C in 5 °C steps. The trend of the average maximum mode share is almost the same in Figure 13 and Figure 14 due to the cold-start emissions. The variation in the maximum mode share due to changes in the temperature is rather small: +/−0.3 pp for HC, +/−0.9 pp for CO, +/−9.1 pp for NO_x_, +/−13.3 pp for PM_10_, +/−0.0 pp for PM_10_ (non-exhaust), +/−1.4 pp for CO_2_ (total), and +/−1.4 pp for CO_2_e WTW.

### 3.13. Variation in the Length of Trips by Customers (Figure 15)

The trip of the customer to the locker is modelled as part of another 1–2 km, 2–3 km, 3–4 km, or >20 km long trip. If the trip to the locker is longer than these km ranges, no maximum mode share has been calculated.

While the effect in regard to cold-start emissions which can be seen in the previous example (Figure 13 and Figure 14) can also be seen in Figure 15, the effect is much lower. The average maximum mode share is similar in Figure 13, Figure 14 and Figure 15 in larger operating areas. However, the average maximum mode share is approximately twice as high in smaller operating areas in Figure 15 compared to Figure 13 and Figure 14. The variation in the maximum mode share due to changes in the customer trips length is rather small: +/−1.0 pp for HC, +/−2.1 pp for CO, +/−3.0 pp for NO_x_, +/−7.6 pp for PM_10_, +/−0.0 pp for PM_10_ (non-exhaust), +/−1.1 pp for CO_2_ (total), and +/−1.1 pp for CO_2_e WTW.

### 3.14. Maximum Spread in the Results

Table 4 shows the average coefficient of variance for each simulation and emission factor. The coefficient of variance is largest for most pollutants (i.e., HC, CO, NO_x_, PM_10_) when the delivery van age or vehicle type (i.e., drivetrain) is changed while the customer vehicle stays as is. The coefficient of variance is the smallest (i.e., <0.02) when the gradient is changed for all pollutants but HC. The coefficients of variance of the maximum mode share based on CO_2_ and CO_2_e WTW are similar unless the delivery vehicle type (i.e., drivetrain) or vehicle age is changed. This is due to the total CO_2_ emissions of different drivetrains being much more similar when the WTT emissions resulting from the production of energy for the vehicle are considered as well. Hence, the coefficient of variance is smaller. The PM_10_ (non-exhaust) emission factor of LCV and PMV are the same and only change when the LOS changes. Hence, the maximum mode share based on PM_10_ (non-exhaust) only changes when the delivery to lockers and delivery to customer homes happen under different LOS, e.g., the PM_10_ (non-exhaust) per km is almost double when a vehicle travels at stop-and-go (0.045 g/km) compared to free-flow (0.026 g/km).

**Figure 16:**
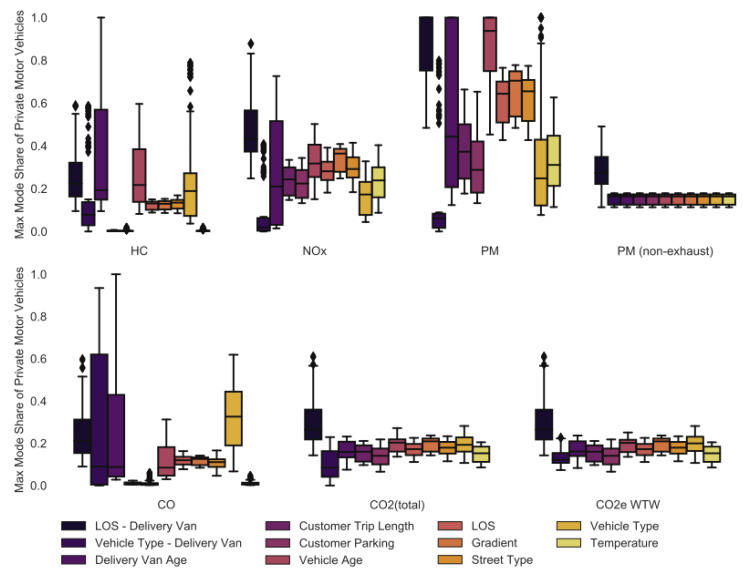
Variance in the maximum mode share.

Figure 16 shows a boxplot for all pollutants and simulations, which highlights the problem of comparing the emissions produced by home delivery and delivery to parcel lockers: The results largely depend on the parameters chosen in the simulation. In fact, any result can be achieved through either changing the pollutant or the parameters of the emission simulation. Hence, it is concerning that small changes in the assumptions of the emission simulations have a significant effect on whether delivery to parcel locker or home delivery is better. This also shows that simply using an average emission factor as all studies did in Table 1 might not be representative of the real world. It is crucial to perform a sensitivity analysis specifically for the relevant parameters in the simulated case.

Almost any maximum mode share based on PM_10_ can be reached when the assumptions in the simulation are changed. A similar picture can be seen for NO_x_, HC and CO emissions, where any maximum mode share between 0% and 60% can be reached. Only the range of possible maximum mode shares based on PM_10_ (non-exhaust) and CO_2_ emissions is small (i.e., the maximum mode shares rarely exceeds 30%).

## 4. Conclusions

Several solutions to the last-mile delivery problem have been proposed in the literature including innovative vehicles, parcel lockers, collaborations, policies, as well as transport management and routing optimization [55]. This paper focuses on evaluating the emissions produced by home delivery with delivery to parcel lockers. This paper highlights two things: the importance of sensitivity analysis of emission simulations as well as the large effect of the customer trip to parcel lockers on the total emissions. When comparing the emissions of home delivery and delivery to parcel lockers, the fraction of customers picking up a parcel from a parcel locker by car has to be kept low as otherwise, home delivery would result in fewer emissions. If only CO_2_ emissions and PM_10_ (non-exhaust) are evaluated, the mode share for cars needs to be less than 20% and 15%, respectively, which is significantly lower than the share of people stating that they would drive a car to pick up a parcel (43–70%) [4,21,26,27,28]. However, the maximum mode share for cars based on PM_10_ (exhaust) emissions is usually approximately 60%. This can vary significantly depending on the assumptions made in this study and can exceed 100%. For example, when the traffic flow for home delivery and customer trips is worse than for the delivery trip to lockers (i.e., delivery at night time), the customer can always pick up a parcel by car and parcel locker delivery would still be better. For NO_x_, the maximum mode share for cars ranges between 20% and 40%.

While it is vital to minimize CO_2_ emissions, only considering CO_2_ emissions fails to appreciate the impact of other pollutants on the environment. Firstly, PM_10_ and NO_x_ are named as one of the most harmful pollutants in cities in Europe [2]. Secondly, the PM_10_ emission factor and NO_x_ emission factor is 4- and 2-fold larger for LCV than for passenger vehicles, respectively, in the default configuration, while all other pollutants are similar (±20%). Thus, a reduction in VKT of the delivery van by delivering to lockers has a much larger effect on PM_10_ emissions even if the customer VKT increases.

In the default case, the PM_10_ and NO_x_ emissions, which are two of the most dangerous pollutants to humans in cities [2], can be reduced by delivering to parcel lockers (Figure 3 and Figure 4) even if half or one-third, respectively, of the customers pick up a parcel by car. However, to reduce the CO_2_ and other pollutants (e.g., CO, HC), almost no customers should use a car to pick up a parcel from CDPs. The maximum mode share for customers varies significantly depending on the drivetrain of the delivery vehicle, delivery vehicle age, cold/warm start of the customer pick-up trip, and temperature. Thus, it is important to utilize a detailed emission model relevant for a specific city as well as a sensitivity analysis and not just use average values as in other studies in order to determine insight into parcel delivery strategies that minimize the impact on the environment. This paper also highlights that policy makers should not just believe a simulation that only considers average values. Instead, they should require that simulations include a variety of scenarios and conduct a sensitivity analysis. By doing so, the range of possible results can be appreciated. If only a single value is presented as result, it is unknown how precise the simulated result is.

## Figures and Tables

**Table 1:** Studies about emissions and parcel lockers.

Authors	Pollutants	Locker vs.	Customer Trips	1st Delivery to Lockers	Best	Simulation/Real
Jiang et al. [8]	carbon emissions	Home delivery	No	Yes	Lockers	Simulation
Carotenuto et al. [19]	CO_2_	Home delivery	No	Yes	Lockers	Simulation
Saad et al. [31]	CO_2_	Home delivery	No	Yes	Lockers	Simulation
Mentioned in [6,7,17,32]	CO_2_, fuel consumption	Home delivery	No	Yes	Lockers	Real
Giuffrida et al. [22]	CO_2_e	Home delivery + depot	Yes	Yes	Lockers ^1^	Simulation
Kiousis et al. [33]	CO_2_, NO_x_, PM_10_	Home delivery	Yes	Yes	Lockers	Simulation Vissim
Song et al. [21]	CO_2_	Depot (failed deliveries)	Yes	No	Depends on, e.g., customer mode choice	Simulation
Song et al. [34]	CO_2_	Redelivery to home, or depot	Yes	No	Lockers	Simulation
Edwards et al. [35]	CO_2_	Redelivery to home, or depot	Yes	No	Post office (i.e., Lockers)	Simulation (only average values)

^1^ if customer travels < 0.94 km.

**Table 2:** Parcels in each operating area.

Scenario	0	1	2	3	4	5	6	7	8	9	10	11	12	13	14	15	16	17	18	19
Size (km^2^)	12	20	31	44	55	68	83	96	116	134	154	178	196	219	244	265	298	318	350	372
Parcels per km^2^	16.2	10.3	6.4	4.5	3.7	2.9	2.4	2.1	1.7	1.5	1.3	1.1	1.0	0.9	0.8	0.8	0.7	0.6	0.6	0.5

**Table 3:** Maximum mode share for varying delivery vehicle drive trains (average over all operating areas).

Drivetrain/Fuel Type	HC	CO	NO_x_	PM_10_	PM_10_ (Non-Exhaust)	CO_2_	CO_2_ WTW
petrol (4S)	50%	79%	6%	7%	15%	14%	14%
diesel	11%	9%	34%	67%	15%	19%	19%
electricity	0%	0%	0%	0%	15%	0%	11%
biofuel CNG/petrol	13%	63%	4%	7%	15%	13%	13%
plug-in hybrid petrol/electric	3%	8%	0%	2%	15%	4%	10%
plug-in hybrid diesel/electric	6%	0%	1%	7%	15%	5%	13%

**Table 4:** Average coefficient of variance.

Simulation	HC	CO	NOx	PM_10_	PM_10_ (Non-Exhaust)	CO_2_	CO_2_ WTW
3.10. LOS for Home Delivery/Customer	0.373	0.406	0.239	0.385	0.259	0.287	0.288
3.8. Vehicle Type—Van	1.215	1.200	1.627	1.568	0.000	0.725	0.227
3.7. Delivery Van Age	1.710	1.724	0.847	1.238	0.000	0.200	0.182
3.13. Customer Trip Length	0.013	0.031	0.032	0.189	0.000	0.068	0.068
3.11. Customer Parking	0.671	0.828	0.060	0.222	0.000	0.180	0.180
3.6. Vehicle Age	0.533	0.696	0.218	0.211	0.000	0.079	0.048
3.9. Level of Service (traffic flow)	0.024	0.097	0.103	0.045	0.000	0.085	0.085
3.5 Gradient	0.042	0.024	0.016	0.008	0.000	0.019	0.019
3.4. Street Type	0.057	0.184	0.113	0.040	0.000	0.082	0.082
3.12. Temperature	0.664	0.576	0.288	0.235	0.000	0.067	0.067

## Data Availability

[Address Points] Department of Information Technology and Telecommunications (DoITT). NYC Address Points|NYC Open Data. 2019. Available online: https://data.cityofnewyork.us/City-Government/NYC-Address-Points/g6pj-hd8k (accessed on 5 February 2019). [Population density] Department of City Planning (DCP). Census Demographics at the Neighborhood Tabulation Area (NTA) Level|NYC Open Data. 2018. Available online: https://data.cityofnewyork.us/City-Government/Census-Demographics-at-the-Neighborhood-Tabulation/rnsn-acs2 (accessed on 17 April 2019). [Parcel deliveries] Department of Transportation. Citywide Mobility Survey—Main Survey|NYC Open Data. 2017. Available online: https://data.cityofnewyork.us/Transportation/Citywide-Mobility-Survey-Main-Survey/dd6w-hnq9 (accessed on 13 February 2019). [Bike sharing stations] Motivate. Citi Bike System Data|Citi Bike NYC. 2019. Available online: https://www.citibikenyc.com/system-data (accessed on 8 May 2019). Map data copyrighted OpenStreetMap contributors and available from https://www.openstreetmap.org (accessed on 1 May 2019).
